# Telemedical versus onsite treatment at an orthopaedic university clinic: Study of 280 consecutive patients

**DOI:** 10.1016/j.ocarto.2021.100140

**Published:** 2021-02-15

**Authors:** Pabinger Christof, Lothaller Harald, Leys Nicolas, Dollnig Samuel, Dammerer Dietmar

**Affiliations:** a Medical University of Innsbruck, Christoph-Probst-Platz 1, Innrain 52 A, 6020, Innsbruck, Austria; b University of Music and Performing Arts Graz, 8010 Graz, Leonhardstraße 15, 8010, Graz, Austria; c Medical University of Innsbruck, Dept. of Orthopaedics, Anichstraße 35, 6020, Innsbruck, Austria

**Keywords:** Telemedicine, eHealth, Tele work, Work from home, Distant work

## Abstract

**Objective:**

Compare a telemedical treatment (distant working) with an onsite treatment. Telemedical services have been used frequently in non-surgical disciplines. It remains unclear if orthopaedic outpatients can be treated via telemedicine. We evaluated the diagnostic accuracy and recommended therapy of a mobile healthcare communication app.

**Design:**

We conducted a prospective, double-blind, anonymized clinical study of consecutive outpatients at an orthopaedic department at a university hospital. Patients were treated by an onsite doctor, who then uploaded each patient’s variables (e.g. personal history, clinical findings, radiograph) for evaluation by a telemedical doctor. The telemedical doctor received the information only via app and did not see the patient physically. Both the onsite and telemedical doctors then uploaded their respective diagnosis and suggested therapy, blinded to each other. The patient received treatment from the onsite doctor only: virtual treatment was solely for scientific purposes and had no therapeutic impact.

**Results:**

Among 280 consecutive orthopaedic outpatients (57% female and 43% male), the mean age was 63 years. In 83% of cases, the telemedical diagnosis matched the onsite diagnosis, and in 98% of cases, the telemedical treatment did no harm. In 75% of cases, the onsite and telemedical doctors proposed the same therapy. In 2% of cases, the telemedical therapeutic regimen differed from the onsite treatment and could possibly harm the patient.

**Conclusion:**

The results suggest that diagnosis and treatment via telemedicine seems feasible in the field of orthopaedic surgery and could be an option for telemedical patient interactions (via work from home or virtual interactions).

## 1 Introduction

Epidemiologic changes (e.g. aging, COVID-19) and economic changes (e.g. limited resources, budgetary restrictions) will narrow the gap between healthcare demand and supply in most countries worldwide. Telemedical patient interactions (via work from home or virtual interactions) can be a viable option to meet the growing demand for healthcare worldwide [[1], [2], [3], [4], [5], [6]].

In Western countries with elderly populations, telemedicine can provide high-quality healthcare for more people without the need for more physical space. In economically developing countries, telemedicine can offer medical care in regions with limited services due to insufficient supply of health facilities, widespread and dispersed populations, geographic barriers, climate issues, economic and political factors, and epidemiologic conditions. In this way, telemedicine offers an alternative to building new hospitals.

Attitudes and behaviours toward telemedicine indicate a growing demand for this technology among both patients and medical doctors [7]. The COVID-19 crisis has further highlighted the need for such tools [6,[8], [9], [10], [11], [12]]. Whereas telemedical services and mobile apps already were used since years in radiology, neurology, and internal medicine [2,3,8,[13], [14], [15], [16]], the COVID-19 crisis has required the use of telemedicine and work from home in other disciplines, such as orthopaedics [6,17,18]. Little is known if telemedicine can be used to treat surgical outpatients, triage patients (prehospital management), or indicate diagnoses [14,[18], [19], [20], [21]]. A recent review described telemedicine during COVID-19 in orthopaedics as safe and cost effective and did not find clinically significant differences in patient examination nor measurement of patient-reported outcome measures [1].

As described above usually telemedical settings involve a remote patient and an onsite doctor. Since the orthopaedic university clinic has a strong focus on surgical interventions, this remote patient setup would rather not work in our patients. We therefore went a step back and first wanted to identify, if medical information regarding acute patients in our outpatient unit can be transported via a simple app and if a remote doctor would be able to find the correct diagnosis (and therapy) as compared to the onsite doctor. If this remote working approach would work (what we did not expect), then we could figure out those diagnoses in the future, where no surgical involvement would be necessary (e.g. lower back pain) and develop a patient-doctor-interaction app in the future in order to provide faster help and to take the load off the out-patient unit.

Therefore, the aim of the current study was to evaluate if a mobile healthcare communication app could be used to identify the correct International Statistical Classification of Diseases and Related Health Problems (ICD-10) diagnosis and the correct therapy, or diagnostic related groupings (DRG), for outpatients at an orthopaedic department at a university clinic.

## 2 Material and methods

This prospective, double-blind, anonymized clinical study included 280 consecutive outpatients presenting for any reason at a university orthopaedic clinic in 10 consecutive months (with 28 consecutive patients each month) in 2019. All experimental protocols were approved by the local IRB/ethics 127 committee (Ethikkommission der Medizinischen Universität Innsbruck, Innrain 43, A128 6020 ​129 Innsbruck). The local IRB/ethics committee (Ethikkommission der Medizinischen Universität Innsbruck, Innrain 43, A 6020 Innsbruck) waived the need of informed consent, since the data is de-identified (anonymous and not pseudonymous and not personalized) and therefore concerns the secondary use of existing data. Therefore all methods were carried out in accordance with relevant guidelines and regulations.

Each patient was assigned to an onsite treatment (ONSITE group) and telemedicine treatment (TELEMEDICAL group). All patients served as their own controls. For the onsite treatment, patients were treated as usual by their onsite orthopaedic doctor. For the telemedical treatment, a remote doctor was chosen by random by the app and reviewed anonymized patient data that was uploaded by the onsite doctor to a web application (www.xMEDx.com, Asklepios GmbH, Plüddemanngasse 45, 8010 Graz, Austria. Patient data included the following primary variables, which were uploaded into the app: personal history (anmnesis), clinical findings (physical status), and one attachment (radiograph, MRI, laboratory, or other relevant result). Both the onsite doctor and virtual doctor were orthopaedic specialists and uploaded their diagnosis (ICD-10) and recommended therapy (DRG) then separately to the app, blinded to each other. All patients received onsite treatment only. Virtual treatment was assessed only for scientific purposes and had no therapeutic impact. The telemedical doctor received patient information only via the app, and they did not interact in any way with the patient or with the onsite doctor. Both onsite and telemedical doctors were allowed to use the internet and library and to discuss the case with colleagues.

The onsite treatment was defined as the correct treatment. Thus, the scientific assessment was to determine if the telemedical therapy matched the onsite treatment regarding proposed diagnosis and treatment. To determine whether both answers matched, a senior supervising (and blinded) doctor checked both answers, and an independent statistician compared the two groups.

**Fig. 1:**
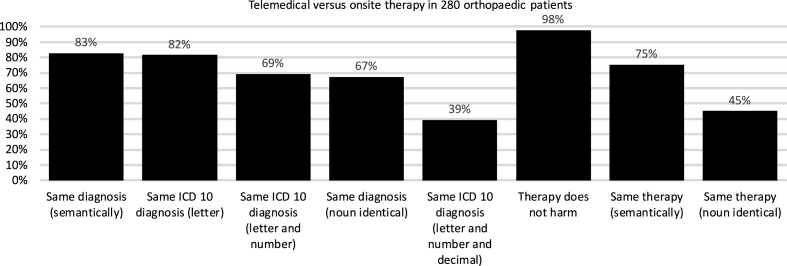
Primary outcome of telemedical versus onsite therapy in 280 orthopaedic patients: The same diagnosis was found in 83% by telemedical means as compared to the on-site treatment. The telemedical proposed therapy did not harm in 98% of all cases.

The following primary outcome parameters were retrieved (Fig. 1 and Table 1):

1. onsite ICD-10 diagnosis (was per definition correct)
2. onsite therapy/DRG (was per definition correct)
3. proposed virtual telemedical ICD-10 diagnosis
4. proposed virtual telemedical therapy/DRG

The following secondary outcome parameters were analyzed (Table 1), since we wanted to assess the interoperability of smartphones and the length of text needed.

5. onset of symptoms (acute/chronic)
6. length of text (number of characters) of anamnesis and status
7. software of the browser
8. type of mobile device/camera/screensize
9. attachment (radiograph/MRI/laboratory result)
10. anatomic region (hip and femur/knee and shin/foot and ankle/head and spine/shoulder and elbow/hand and wrist/other)
11. age/working experience/gender/tenure of the doctors

**Table 1:** Secondary outcome of telemedical versus onsite therapy in 280 orthopaedic patients: Patient with a longer personal history and longer clinical findings had a higher degree of conformity between on-site and telemedical diagnoses and treatments, but this was not a significant finding. No specific anatomic region or onset or type of mobile device has a tendency towards an inferior or better diagnosis regimen.

SECONDARY OUTCOME PARAMETERS		Same diagnosis (semantically)		
Telemedical versus onsite diagnosis		Right	Wrong	p-value
Patients	**280**	**232**	**48**	
Acute onset	24%	81%	19%	**n.s.**
Chronic onset	76%	86%	14%	**n.s.**
Length of text				
Personal history (characters)	245 ​± ​185 (29–1342)	256	220	p ​= ​.07
Clinical findings (characters)	117 ​± ​109 (0–618)	126	120	p ​= ​.08
Software of the browser	>10 different types	**83%**	**17%**	**n.s.**
Type of mobile device	>20 different types	**83%**	**17%**	**n.s.**
Attachment	**100%**	**83%**	**17%**	**n.s.**
Radiograph/MRI	85%	88%	12%	
Laboratory result	2%	88%	12%	
Both	8%	88%	12%	
No attachment	5%	100%	0%	
Anatomic region	**100%**	**83%**	**17%**	**n.s.**
Hip and femur	29%	85%	15%	
Knee and shin	22%	89%	11%	
Foot and ankle	15%	77%	23%	
Head and spine	16%	78%	22%	
Shoulder and elbow	8%	62%	38%	
Hand and wrist	7%	77%	23%	
Other	3%	83%	17%	

Statistical analyses aimed at comparing the ONSITE and TELEMEDICAL groups. Depending on the given variables, either cross-tables with chi-square-tests or *t* tests for paired samples were calculated.

• ICD Diagnoses were analyzed if they were identical regarding (a) the letter, (b) the letter and the number (c) the letter, the number, the decimal.
• Free text diagnoses were analyzed if (a) the same noun or (b) the semantically same meaning (synonymon) was used.
• The telemedical therapy/DRG was analyzed if it would (a) harm the patient and if (b) the same noun or (c) the semantically same meaning (synonymon) was used.

Preconditions (normal distribution of homogeneity of variances for testing differences of means by parametrical test) were considered and kept, if applicable. Results with p-values below .05 were considered significant.

## 3 Results

Among the 280 consecutive orthopaedic outpatients treated, 57% were women and 43% were men. The mean age was 63 ​± ​8 years (range: 18–93). The most common diagnoses were hallux valgus/rigidus, hip arthritis, knee arthritis, meniscopathy, scoliosis, carpal tunnel syndrom, lumbalgia.

### 3.1 Primary outcome parameters

For ICD-10 classification, the telemedical diagnosis used the correct semantic (synonymon) diagnosis in 83% of cases and the same noun in 67% of cases, and the same therapy (correct semantic/synonymon) was proposed in 75% of cases. In most (98%) of cases, the telemedical treatment did no harm. In 2% of cases (six patients), the telemedical therapeutic regimen differed from the onsite regimen and could possibly harm the patient or lead to overtreatment or undertreatment, which was assessed qualitatively by two orthopadic specialists in the following cases. In one instance, a femoral head metastasis was mistaken for a primary tumour (osteoblastoma). In another instance, conservative therapy was recommended instead of operative revision for a postoperative wound infection. In two cases, a hallux valgus was recommended for operative treatment instead of using a conservative approach and in two cases operative treatment was suggested too early from the telemedical doctor (hip replacement and knee replacement were recommended instead of physiotherapy). Fig. 1 shows the results.

### 3.2 Secondary outcome parameters

No statistical significant difference (p>.05) was observed between telemedical and onsite treatments for any of the secondary parameters (onset, length of text in characters, software type, mobile device type, attachment type, anatomic region). Cross-table queries generated no significant or strong correlations between groups. In other words, the diagnoses matched regardless of using a mobile device or desktop computer and regardless of software and browser type. Incorrect telemedical diagnoses were not related to insufficient quality of medical images or software or browser issues, regardless of the device/camera and screen size used.

Longer personal history (p ​= ​.07) and longer descriptions of clinical findings (p ​= ​.08) were associated with higher likelihoods of receiving a correct diagnosis. In 5% of cases, the clinical symptoms were clear and no image was uploaded, and the telemedical diagnosis was correct in all of these cases. Among the 76% of patients with chronic onset, 86% of telemedical diagnoses matched the onsite ones. Among the 24% with acute problems, 81% of telemedical diagnoses matched the onsite ones. Results were comparable for all anatomical regions and joints. Diseases of the shoulder and elbow had the lowest number of correct answers (62%), but the sample size (8% of cases) was too small to be statistically significant in a multivariate correlation analysis. We observed statistically significant differences for the parameter “ICD-10 diagnosis (letter and value).” For example, “M23 internal derangement of the knee” has 60 sub-diagnoses and showed a significantly low number of only 25% correct telemedical answers (p ​= ​.02), compared with all other diagnoses. In contrast, forefoot diagnoses “M.20.1 hallux valgus” and “M.20.2 hallux rigidus” were correctly diagnosed in 100% of cases. Table 1 shows the results.

Age/working experience/gender/tenure of the doctors had no influence on outcome in a multivariant analysis.

## 4 Discussion

In our study, 76% of patients had chronic symptoms for months and therefore no urgent need for hospital admission. We found that the telemedical ICD-10 diagnosis matched onsite diagnoses in 83% of cases, and in 75% of cases, the suggested therapies in the telemedical setting were similar to those in onsite therapy. In 98% of cases, the telemedical therapy did no harm, and in the other 2%, the telemedical therapeutic regimen differed from onsite treatment and could possibly harm or lead to an over- or undertreatment of the patient. Although concerning, these results may not be unique to telemedicine, as other research shows that at least one-third of all published onsite medical practices are not evidence-based and may be worse than doing less or nothing [22]. Consecutively a pre-hospital telemedical triage (e.g. with the general practitioner) could facilitate the access to care for orthopaedic patients, as described in other medical disciplines [[2], [3], [4],^14^,^19^].

To the best of our knowledge, this is the first study prior to COVID-19 to assess the feasibility of telemedical patient interaction and the possibility of distant working (e.g. work from home or virtual interaction) in an orthopaedic setting at a large university hospital using a prospective, consecutive, double-blind design. Thus far, telemedical services have been found to be effective mainly in non-orthopaedic disciplines [9,[23], [24], [25], [26], [27]] or as pilot studies in specific patient groups (telerehabilitation) [21,23,24,[28], [29], [30], [31]].

Our study showed, that in some diagnoses like “painful hallux valgus” a telemedical answer (“hallux operation”) can be easily obtained. However, other diagnoses (like “meniscal damage”) will result in a lower degree of treatment conformity, since several different treatment choices are possible. Yet, 30% of the population in industrialised countries are affected by arthritis and the share of orthopaedic patients will exponentially increase in the future [32,33].

Inequality in access to (orthopaedic) care is a worldwide phenomenon [34,35]. The use of telemedicine in different medical specialties are well-known, with proven effectiveness, efficacy, accuracy, and usefulness [9,[23], [24], [25], [26], [27]]. Thus, telemedicine has the potential to improve access to healthcare in rural areas around the world [4,5].

Recently, COVID-19 has required adjustments to ensure the safety of both healthcare staff and patients. As a result, the use of telemedicine has increased worldwide since it also can protect the well-being of the healthcare workforce, which is crucial for successful health systems [36]. Telemedicine platforms thus can be ideal for managing healthcare challenges in response to global infectious disease outbreaks [37]. But usefulness, compliance and the attitude towards telemedicine is discussed controversially in both, patients and health care professionals [7,11,30,38,39].

Our study measures outcome, when doctors put data in a medical app. This is rather uncommon and might be used for work at home. Another usecase is, when patients themselves use an orthopaedic app on their own. We assume, that this would lead to a worse degree of conformity and hence additional data would be useful to process such a request (step count, gait analysis, medical images, …)

Up to now the Austrian healthcare system is perceived as one of the best worldwide, as it provides almost fully covered high-quality care with free choice of providers and unrestricted onsite access to general practitioners, specialist physicians, and hospitals [[40], [41]]. [[,^41^] Nowadays, medical apps serve as important sources of information for many doctors, and 74% of Austrian doctors use medical apps on a daily basis [42]. Our results showed that an orthopaedic outpatient app might be useful for telemedical patient interaction and the possibility of distant working (e.g. work from home) or as a triage tool prior to visiting the outpatient clinic.

Our study has several limitations that must be considered in regard to real-life implementation. First, current telemedicine technology precludes manual examination. Second, although some patients with restricted mobility might benefit from the accessibility of telemedicine, technical issues might hinder use among those with certain impairments (e.g. poor vision, coordinative problems, low computer knowledge). In our study, the use of the telemedical app was intuitive, and no additional support was necessary. However, we did not assess usability among patients. Third, the use of different end-user devices, operating systems, and browsers is challenging for software developers. In our study, we therefore used a web application instead of a native app to improve the ease of use. Although we found no significant constraints or confounders regarding screen size, operating system, type of browser, type of camera, or other variables, we did not assess several other conditions that could affect the quality of telemedical care, such as lighting conditions, internet speed, and so on. Finally, the use of an app prior to admission could generate supply-induced demand, and this may be more harmful than beneficial [43,44] Therefore, the findings of this study are subject to several potential sources of bias that should be noted as limitations.

Telemedicine will never replace conventional medicine. However, blended care involving telemedicine and conventional medicine can enhance future medical care. Prior research indicates that the relationship between telehealth systems and physical medical services is mutually beneficial [9,[23], [24], [25], [26], [27]]. Telemedical consultation seems to work in other disciplines, and it shows promise in the field of orthopaedic surgery.

## Author contributions statement

PC: research design, analysis, drafting and revision. LH: analysis and interpretation. SF: revision and interpretation. LN: analysis and interpretation. DS: analysis and interpretation, acquisition of data. DD: revision and interpretation. All authors have read and approved the final submitted manuscript.

## Declaration of competing interest

Nothing to declare.
